# Sensory Evaluation and Model Prediction of Vacuum-Packed Fresh Corn during Long-Term Storage

**DOI:** 10.3390/foods12030478

**Published:** 2023-01-19

**Authors:** Yilin Li, Kui Zhong, Xue Wang, Houyin Wang, Yongjiu Zhang, Bolin Shi, Huarong Luo, Lei Zhao, Shilong Jiang, Sisi Wang

**Affiliations:** 1 Heilongjiang Feihe Dairy Industrial Co., Ltd., Qiqihar 164800, China; 2 China National Institute of Standardization, Beijing 100191, China

**Keywords:** fresh corn, vacuum packaging, long-term shelf-life, sensory quality, kinetic model

## Abstract

The postharvest shelf life of fresh corn largely depends on the packaging method and storage temperature. This study investigated the effect of vacuum packaging (VP) with high-barrier (HB) or ordinary (OR) nylon/nylon/polypropylene (PP) composite films and the impact of storage temperature (4, 25, and 38 °C) on the shelf life of fresh corn. The sensory quality and color changes of the corn were evaluated, indicating a significant improvement in the glossiness (GL), sourness (SO), and color changes compared to corn packaged using OR films. The results showed that the HB films preserved corn freshness under refrigerated and normal temperature storage conditions, delaying color changes and SO development. A shelf-life model was established based on the Arrhenius equation. The predicted values of the corn at different temperatures were compared with the experimental data, indicating that the model could accurately predict the shelf life. The shelf life observed via sensory evaluation was more than 50% shorter than the results obtained by instrumental measurements. Therefore, sensory evaluation could be applied to determine shelf life and avoid food waste.

## 1. Introduction

Fresh corn is a nutritious food product that is low in fat and calories and high in fiber. It is highly popular with consumers due to its tender mouthfeel and rich fragrance. The main types of corn suitable for fresh consumption include sweet corn (*Zea mays* L. *saccharata Sturt*) and waxy corn (*Zea mays* L. *certaina Kulesh*). Sweet corn (SC) is produced via recessive mutation of one or several endosperm genes (*su*, *sh*, *bt*, and *se*) of common corn species, inhibiting starch synthesis and increasing the soluble sugar content of the endosperm, rendering it significantly sweeter than other maize [1]. However, waxy corn (WC) is derived via recessive mutation of the starch synthase waxy1 (*GBSS-Ⅰ*) gene located on chromosome 9 of common species. This causes a sudden rise in the amylopectin content in the endosperm, increasing the waxy properties and viscosity [2].

Fresh corn is a living organism exhibiting strong postharvest biological activities due to its nutrient richness and moisture content. Therefore, a series of physiological and biochemical changes occur when stored under natural conditions, leading to a decline in quality. Corn can be stored at 0 °C for up to 6–8 d, at 5 °C for 3–4 d, and at 10 °C for 2 d to maintain optimal quality [3]. Since the popularity of fresh corn necessitates a continuous market supply, postharvest preservation is essential. Various methods are used to extend the shelf life of food, including quick-freeze preservation [4,5], modified atmosphere packaging [6,7], and vacuum packaging (VP) [8,9,10]. Considering the packaging costs and convenience of storage and transportation, VP is the most commonly used commercial technique [11], theoretically requiring a 100% vacuum state, that is, a complete absence of air, water vapor molecules, or gas. Therefore, the appropriate packaging material is vital for achieving optimal preservation. Rodov et al. [12] and Pal et al. [13] used single-layer PVC film, perforated multilayer polyolefin film, micro-perforated film, unilamellar polypropylene (PP), bilamellar PP/polyethylene terephthalate (PET), and trilamellar PP/PET/aluminum foil (Al) composite material to extend the shelf life of fresh corn, and compared their freshness preservation abilities. The results indicated a significant correlation between the water vapor transmission rate (WTR) and oxygen transmission rate (OTR) of the material and the freshness preservation effect. Therefore, multilayer packages with high-barrier (HB) properties, such as EVOH, metal oxide-coated PET, and oxygen scavenger-embedded structures, have been developed to improve efficacy. High-barrier (HB) nylon/nylon/PP is a new composite material used in a variety of food preservation packaging materials. However, the ability of these packaging materials and ordinary (OR) nylon/nylon/PP materials to improve the shelf life of fresh corn is unknown.

The sensory characteristics of food are essential quality indicators and play a critical role in consumer product expectations, choices, purchases, preferences, and acceptability [14,15]. However, most studies investigating quality changes during storage have focused on compound changes [16,17,18,19,20], respiration and transpiration [21,22], and microbial growth [23]. Furthermore, since shelf-life predictions are based on substantial changes and microbial proliferation, one of the main objectives is measuring and ensuring the freshness of the product to promote consumer acceptance [24]. Compound changes are not necessarily reflected by sensory characteristics. Therefore, this study prepared two composite films (HB and OR materials) to extend the shelf life of two main types of fresh corn based on their sensory qualities and assessed the benefits for the food packaging industry. The characteristic sensory quality indexes were measured during storage, including fullness (FU), glossiness (GL), stickiness (ST), corn flavor (CF), sourness (SO), and sweetness (SW). Furthermore, the color of the samples was also measured using a colorimeter. A shelf-life model was established to provide theoretical guidance for the commercial production of vacuum-packaged fresh corn.

## 2. Materials and Methods

### 2.1. Packaging Material

This study used two kinds of nylon/nylon/PP composite film packaging materials: HB and OR composite films. The thickness of one side of the composite film was 100 μm. The OTR values of the HB and OR composite films were 0.3 and 28 cc/m^2^/day at 1 atm, respectfully, while the WTR values were 3.5 and 5 g/m^2^/day, respectively.

### 2.2. Fresh Corn Raw Materials and Packaging

Two kinds of fresh corn, including sweet corn (SC) and waxy corn (WC), were procured from a corn plantation in Qiqihar, Heilongjiang Province, China. The two varieties are available in the Chinese market, namely Aofulan (sweet corn, Validation No. 20210632) and Jinnuo 262 (waxy corn, Validation No. 2015054), and their detailed information can be found at http://a-seed.cn/ (accessed on 5 July 2021). The corn was harvested at the milk ripening stage (25–28 d after silking) on the same day. Cobs of uniform size with no mechanical damage were chosen and transported immediately to the local fresh corn processing factory. After removing the leaves, filaments, and stem tips, the fresh corncobs were cleaned to remove soil and dirt, after which they were immersed in hot water at 95–98 °C for 5 min and then cooled quickly to room temperature. The corn was air-dried at ambient temperature and vacuum-packaged with HB and OR composite films. The packaged corn samples were sterilized at 121 °C for 30 min and then immediately cooled to room temperature under running water. Finally, four types of packaged fresh corn products were obtained for the storage experiment, including SC packed in HB composite film (SC-HB), SC packed in OR composite film (SC-OR), WC packed in HB composite film (WC-HB), and WC packed in OR composite film (WC-OR).

### 2.3. Sample Preparation

Five corncobs from twelve treatments (four kinds of corn samples, three different temperatures) were randomly selected. After removing the packaging, the corn was placed in a Demashi steam box (each sample was placed in a separate steamer drawer) and steamed for 20 min. The samples were removed and drained of excess water, after which approximately 3 cm was removed from each corncob head and tail. The middle section of the corncob was cut into 3 cm segments and placed on a white disk for heat preservation in a 50 °C thermostatic cabinet until use. Sensory evaluation was conducted within 2 h of corn sample preparation. The samples were labeled with random three-digit numbers and presented to each assessor in arbitrary order.

### 2.4. Experimental Settings

The experiment was based on standard GB/T 38493. Each packaged fresh corn sample was divided into three equal groups and stored at 4, 25, and 38 °C, respectively. The storage temperature was monitored by sensors to ensure its stability throughout the experiment (±2 °C). The samples stored at 38 °C were measured every 15 d, while those stored at 4 and 25 °C were observed every 30 d. The total experimental storage period lasted for seven months.

### 2.5. Sensory Evaluation

#### 2.5.1. Sensory Panel

The sensory panelists were selected, trained, and monitored according to ISO 5496 and ISO 8586 standards. The ten selected panelists (five females and five males with a mean age of 22.4 ± 3.4 years) were required to be healthy, not use drugs or smoke, have no taste/olfactory disorders, be free of chronic diseases such as hypertension or heart dis-ease, and not be allergic to corn and related products. In addition, they had to recognize and screen the thresholds of basic taste and smell and be able to evaluate and discuss sensory attributes. Furthermore, they were also required not to have experienced any cold symptoms for at least one week before the sensory evaluation. The sensory panel training lasted for more than 8 h and included the identification, description, and rating of CF and familiarity with evaluation procedures. The sensory evaluation only commenced when the evaluation panel displayed excellent repeatability and consistency.

#### 2.5.2. Sensory Evaluation

The sensory quality evaluation of corn samples was conducted in a standardized sensory test room (ISO 8589), which consisted of an adequately air-conditioned environment, with the temperature in the booths controlled at approximately 25 °C. Six fresh corn sensory descriptors were developed via a round table discussion according to the ISO 5492 standard and NY/T523-2020, which included FU, GL, ST, CF, SO, and SW. Furthermore, the sensory attribute intensities of the fresh corn samples were evaluated using a 10-point line scale, denoting an interval quantitative response scale ranging from 0 (none) to 10 (extremely strong), signifying low to high intensity [25]. Each panelist was placed in a separate booth and required to evaluate the sample and respond to the questions independently.

Twelve corn samples (four kinds of corn samples, three different temperatures) were evaluated over two sessions to prevent sensory physiological fatigue. Each panelist rated the six sensory attributes using the 10-point scale, evaluating the next sample only after no residual taste remained in their mouth. The panelists were allowed to drink purified water to prevent an aftertaste. The sensory evaluation was approved by the Ethics Committee of Tsinghua University and all panelists provided informed written consent before the experiments.

### 2.6. Color Value

The color of the fresh corn samples was measured using a MiniScan Chroma meter (HunterLab, Memphis, Tennessee, USA). The reflectance values of ten replicates was recorded for each set of samples. The color values were expressed as brightness (L*), red-green (a*), and yellow-blue (b*) values. The total color difference (Δ*E*) was calculated according to the equation below [26]:

(1) $$\Delta E=\sqrt{{\left(L^{*}-L_{0}^{*}\right)}^{2}+{\left(a^{*}-a_{0}^{*}\right)}^{2}+{\left(b^{*}-b_{0}^{*}\right)}^{2}}$$

where L*, a*, and b* are the brightness, red-green, and yellow-blue values of the stored samples, respectively, and $L_{0}^{*}$, $a_{0}^{*}$, and $b_{0}^{*}$ are those of the initial sample values.

### 2.7. Establishment of the Shelf-Life Model

Zero- and first-order kinetic models were used to construct the sensory and color quality reaction kinetic models of the fresh corn samples, while the Arrhenius model was employed to investigate the effect of temperature [27].

(2) $$\text{Zero-order}\;\mathrm{response}\;\mathrm{model}:\;C=C_{0}+kt$$

(3) $$\text{First-order}\;\mathrm{response}\;\mathrm{model}:\;C=C_{0}e^{kt}$$

(4) $$\text{Zero-order}\;\text{shelf-life}\;\mathrm{model}:\;t=\frac{C-C_{0}}{k_{0}e^{\frac{-E_{a}}{RT}}}$$

(5) $$\text{First-order}\;\text{shelf-life}\;\mathrm{model}:\;t=\frac{ln\frac{C}{C_{0}}}{k_{0}e^{\frac{-E_{a}}{RT}}}$$

(6) $$\mathrm{Arrhenius}\;\mathrm{equation}:\;lnk=lnk_{0}-\frac{E_{a}}{RT}$$

where *C_0_* refers to the initial sample value, *C* denotes the value after storage *t* period, *k* and *k_0_* are the reaction rate constants, *Ea* (J/mol) is the activation energy, *R* (8.3144 JK^−2^mol^−1^) is the molar gas constant, *T* is the absolute temperature in degrees Kelvin (K), and *t* is the sample storage time.

### 2.8. Statistical Analysis

Excel was used for data collection, and IBM SPSS Statistics 19.0 (IBM Corp., Armonk, NY, USA) was employed for the analysis of variance and Duncan’s test, while the significance level was set at 0.05. Origin 2019 software (OriginLab Corporation, Northampton, MA, USA) was used to create the charts and establish the model.

## 3. Results and Discussion

### 3.1. Sensory Profiles

**Figure 1:**
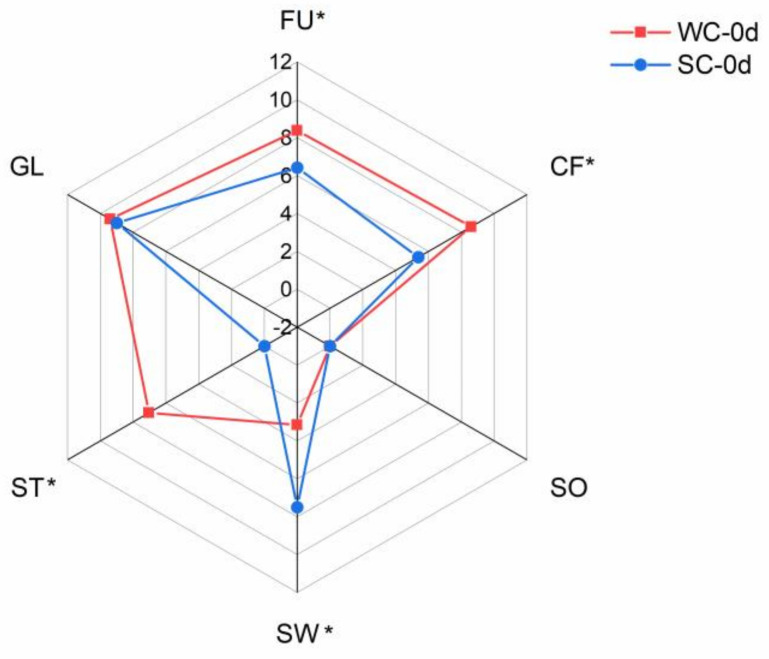
The sensory profile description of two fresh corn sample categories. * indicates that the characteristic indexes significantly differ between the two samples (*p* < 0.05). ANOVA was used to analyze differences between groups. WC-0d indicates waxy corn stored for 0 days. SC-0d indicates sweet corn stored for 0 days. FU, CF, SO, SW, ST, and GL indicate that the characteristic sensory qualities of fullness, corn flavor, sourness, sweetness, stickiness, and glossiness, respectively.

Figure 1 shows the sensory profiles of the two kinds of fresh corn samples at 0 d, presenting different sensory characteristics. The fresh SC samples displayed typical sensory attributes in SW, while the fresh WC samples exhibited higher intensity scores for ST and CF. Both the SC and WC samples presented high GL scores, while WC showed higher intensity scores for CF and FU than SC (*p* < 0.05).

### 3.2. The Sensory Qualities of the Fresh Corn Samples during Storage

The SW, FU, ST, and CF sensory results of the packaged corn samples over the seven-month period are presented in Supplementary Figure S1. No significant (*p* < 0.05) changes were evident in these four sensory attributes at the low temperature treatments (4 and 25 °C) throughout the storage period. In addition, the scores of these four sensory indicators fluctuated within a certain range, but there was no significant (*p* < 0.05) difference between the beginning and end of storage. These results highlighted the excellent freshness preservation effects of VP combined with high-temperature-short-time (HTST) sterilization on the texture and flavor attributes of the fresh corn samples during the storage period, which was consistent with previous reports [13,28]. VP is an effective method for storing and preserving fresh fruit and vegetables [29]. This technique inhibits microbial growth and restricts enzyme activity by reducing the oxygen content in the bag. It has been widely used to maintain the quality of fresh agricultural products [30]. No significant changes were apparent in the soluble sugar and starch contents of the fresh corn samples throughout the storage period (data not shown, data is presented in Supplementary Tables S2–S7), further demonstrating that combining VP and HTST sterilization effectively extended the shelf life of the fresh corn samples. For this reason, it has been used extensively for the cost-efficient quality preservation of fresh agricultural products.

**Figure 2:**
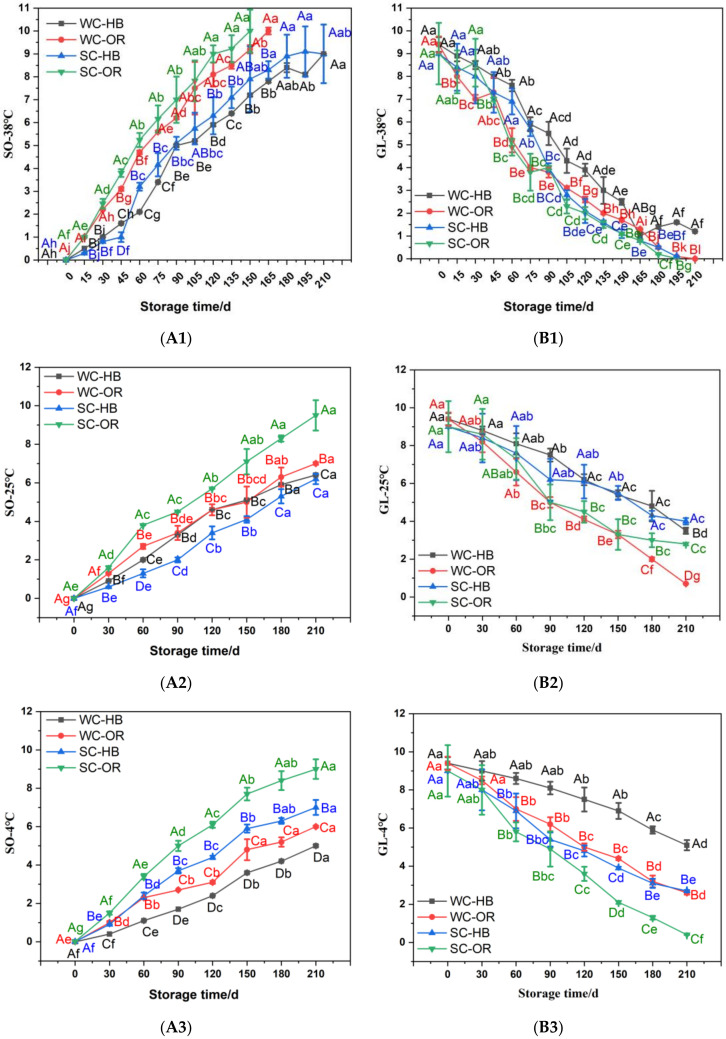
The SO (**A1**–**A3**) and GL (**B1**–**B3**) of fresh corn packaged with HB and OR composite films at three storage temperatures from 0 to 210 d. Different capital letters (A–D) indicate significant differences (*p* < 0.05) between different treatments at the same storage time. Different lowercase letters (a–j) indicate significant differences (*p* < 0.05) between different storage times for the same treatment. Duncan’s test was used to analyze differences between groups. WC-HB indicates waxy corn packaged with high-barrier nylon/nylon/PP composite film. WC-OR indicates waxy corn packaged with ordinary nylon/nylon/PP composite film. SC-HB indicates sweet corn packaged with high-barrier nylon/nylon/PP composite film. SC-OR indicates sweet corn packaged with ordinary nylon/nylon/PP composite film. SO (**A1**–**A3**) and GL (**B1**–**B3**) indicate the characteristic sensory qualities of sourness and glossiness, respectively.

As shown in Figure 2A, the SO of the fresh corn increased at different temperatures. The SO values of the fresh corn samples packaged with HB films were lower than those packaged with OR films. In this study, an SO score higher than 8 indicated that the sample was inedible. Except for SW-OR, the other three treatments exhibited lower SO values (<8) throughout the seven-month storage period at 4 and 25 °C. However, the SO values of the fresh corn samples packaged with HB and OR films increased to 8 when stored at 38 °C for 120 and 165 d, respectively. Therefore, the rancidity rates of the samples packaged with HB films were lower than those packaged with OR films at the three storage temperatures, indicating that the HB films displayed a certain freshness preservation effect under refrigerated and normal temperature storage conditions. Furthermore, the fresh SC samples exhibited higher SO values than the fresh WC samples during the same treatment, which was related to the soluble sugar and water contents [31].

GL is an important quality characteristic during the sensory evaluation of fresh corn and corn products. The GL values at different treatments are shown in Figure 2B. The GL values of fresh corn samples declined as the storage time was extended. In this study, a GL score lower than 4 indicated that the sample was unacceptable. The GL values of the fresh corn samples decreased to 4 after approximately 120 d of storage at 4 and 25 °C. High temperature promoted a decline in the GL score, decreasing the value to 4 after approximately 90 d of storage at 38 °C.

The browning reaction was observed in all treatment groups. The degree of browning of the fresh corn samples packaged with HB films was lower than those packaged with OR films (*p* < 0.05). Fresh WC exhibited a lower degree of browning than SC exposed to the same treatment. Food browning is generally divided into enzyme-dependent and -independent browning [32]. The browning of the fresh corn samples in this study was primarily caused by non-enzymatic browning, which was related to the Maillard reaction, and was extremely sensitive to temperature, oxygen content, and water content [32,33]. The fresh SC displayed a higher degree of browning than the fresh WC exposed to the same treatment, indicating the presence of the Maillard reaction during storage. A higher degree of browning was evident at 38 °C, while no significant differences were observed between the samples exposed to 4 and 25 °C (*p* < 0.05). Therefore, HB film and lower storage temperatures can inhibit the browning of fresh corn samples.

### 3.3. Color Values

**Figure 3:**
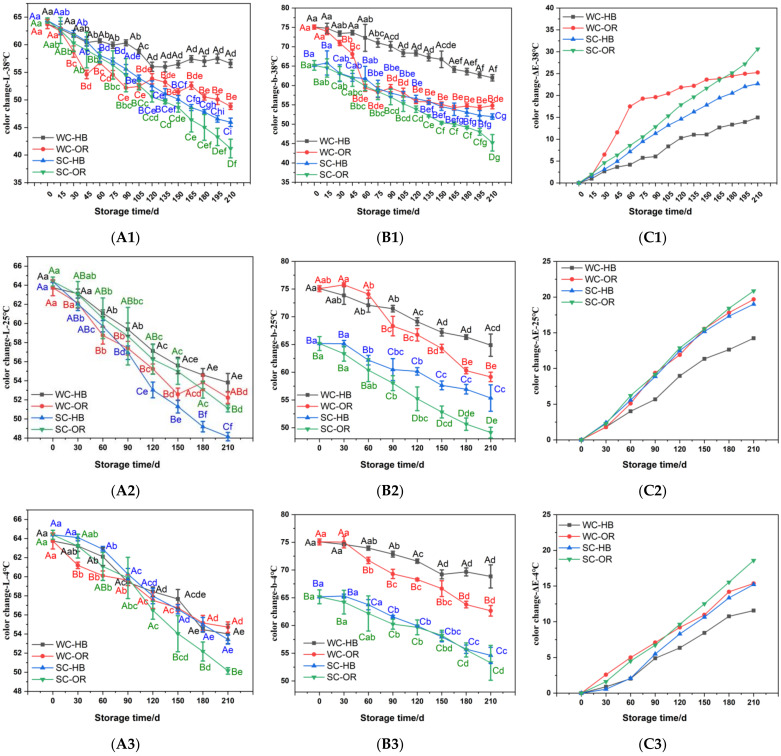
The L* (**A1**–**A3**), b* (**B1**–**B3**), and Δ*E* (**C1**–**C3**) color values of the fresh corn packaged with HB and OR composite films at three storage temperatures from 0 to 210 d. Different capital letters (A–D) indicate significant differences (*p* < 0.05) between different treatments at the same storage time. Different lowercase letters (a–i) indicate significant differences (*p* < 0.05) between different storage times for the same treatment. Duncan’s test was used to analyze differences between groups. WC-HB indicates waxy corn packaged with high-barrier nylon/nylon/PP composite film. WC-OR indicates waxy corn packaged with ordinary nylon/nylon/PP composite film. SC-HB indicates sweet corn packaged with high-barrier nylon/nylon/PP composite film. SC-OR indicates sweet corn packaged with ordinary nylon/nylon/PP composite film. L* (**A1**–**A3**), b* (**B1**–**B3**), and Δ*E* (**C1**–**C3**) indicate that the color values of brightness, yellow-blue, and the total color difference calculated according to the color values, respectively.

Color is an important factor that affects consumer acceptance, preference, choice, and purchase of products. The L* and b* values of the fresh corn samples decreased at the three treatment temperatures (Figure 3A,B). Similar results were observed in many reports involving fresh-cut pineapple [34] and fresh corn [13,19,35], in which the L* and b* values decreased gradually over time, leading to sample darkening and were further characterized by a reduced yellow color. The color reduction could be ascribed to the browning reaction, indicating that the color of the corn became darker and less yellow with extended storage time.

The Δ*E* value refers to the color value difference [36]. As shown in Figure 3C, the Δ*E* values of the fresh corn samples increased during the three treatments. The Δ*E* value changes became more distinct in the corn samples stored at 38 °C compared with those (*p* < 0.05) stored at 4 and 25 °C. Reports have shown that high temperatures increase pigmentation, causing visible color changes in fruits, vegetables, and related products, especially beverages [37]. Packaging with HB films reduced the ΔE values of the fresh WC compared with OR films. Therefore, HB films and lower storage temperatures can inhibit color changes in fresh corn samples.

### 3.4. The Shelf-Life Prediction Models of the Fresh Corn

Distinct differences were evident between the GL, SO, and color values of the fresh corn throughout the storage period. Therefore, these three indices were used to establish shelf-life prediction models of the fresh corn packaged with HB and OR films. A larger coefficient of determination (R^2^) generally presents a more accurate fitting equation. This study used a zero-order equation with a larger R^2^ to establish the GL, SO, and color kinetic models. The equations are shown in Supplementary Table S1.

Furthermore, the shelf-life prediction models of the fresh corn were established according to the Arrhenius equation. As shown in Table 1, the R^2^ of most prediction models exceeded 0.90, indicating a more accurate degree of model fitting [23]. In this study, scores of 4 and 8 were considered the ultimate limit values of the GL and SO shelf-life models, respectively. Furthermore, the Δ*E* value of the corn sample with a GL value of 4 was set as the unacceptable color value limit. Therefore, fresh corn samples with an SO value exceeding 8 or a GL value below 4 were considered unacceptable.

The predicted shelf-life value of the fresh corn at different temperatures was calculated and compared with the experimental data, as shown in Table 2. The models accurately predicted the shelf life when the relative error (RE) did not exceed 20%. As shown in Table 2, the RE between the predicted shelf-life values and the experimental values were below 20% during all the treatments, except for the SO-based shelf life for fresh WC at 4 °C, the GL-based shelf life for fresh SC at 38 °C, and the GL-based shelf life for fresh WC at 25 °C. The results indicated that these models could be used to predict the shelf life of fresh corn at different temperatures.

The SO-based shelf life values of the fresh corn were 50% to 200% longer than those of the color-based shelf life values, suggesting that the unacceptability of the fresh corn could primarily be attributed to the color change rather than the SO. Consumers often reject food (including fruits and vegetables) because of its appearance [38]. Although spoilage can be characterized based on product changes that render it unacceptable to consumers from a sensory point of view [39], academia, research organizations, and authoritative regulatory agencies indicate that product deformation may not necessarily be proof that it is bad or unsafe to consume [40,41]. Therefore, it is necessary to restrict the browning in postharvest fresh corn as much as possible to prolong its commercial value and avoid food waste. The GL value observed via sensory evaluation indicated a shorter shelf life than that indicated by the color changes measured by the MiniScan Chromameter. Therefore, sensory evaluation is necessary to assess the shelf life and cannot be replaced by physical and chemical analysis.

Moreover, the shelf life of the corn packaged with HB films was considerably longer (10–50%) at all storage temperatures than the samples packaged with OR films and more distinct at a high temperature (38 °C). Consequently, it can be concluded that HB films effectively extended the shelf life of fresh corn compared to OR films. The most significant difference between the two materials was evident in the OTR, while no noticeable variation was apparent in the WTR. This finding indicated that the oxygen concentration played a substantial role in fresh corn spoilage.

A comparison involving two packaging methods and three storage temperatures indicated that 4 °C storage and HB film packaging were most successful for the preservation of fresh corn.

## 4. Conclusions

Compared to OR films, using HB films to vacuum-pack fresh corn samples showed an excellent ability to preserve postharvest freshness due to the excellent gas barrier properties. All four treatments yielded significant changes in the GL, SO, and color change indexes at different storage temperatures. Low-temperature storage (4 °C) extended the shelf life by 20–100% compared to high-temperature storage (38 °C). Therefore, low-temperature storage and HB film effectively delayed the GL, SO, and color change indexes in the fresh corn. Moreover, the shelf life determined according to color was 50–200% shorter than that based on SO, while the GL value identified via sensory evaluation indicated the shortest shelf life. Therefore, a shorter shelf life was observed based on the sensory characteristics. Consequently, using sensory evaluation to predict the shelf life of products can effectively prevent food waste.

## Figures and Tables

**Table 1:** The fitting results of the kinetic models at different temperatures and the predicted shelf-life results.

Sample	Characteristic Index	Storage Method	R^2^	K_0_	Ea/kJ/mol
SC	Color changes	HB	0.980	1.974	7452
		OR	0.850	4.488	9110
	GL	HB	0.894	6.669	13,027
		OR	0.999	0.886	7568
	SO	HB	0.852	1.226	8609
		OR	0.941	15.99	14,371
WC	Color changes	HB	0.946	0.400	4355
		OR	0.997	4.423	9440
	GL	HB	0.973	13.579	15,083
		OR	0.941	0.271	4736
	SO	HB	0.948	3.905	11,744
		OR	0.995	31.003	16,191

Note: R^2^ denotes the coefficient of determination of the models, K_0_ refers to the pre-factor, and Ea/kJ/mol is the activation energy. RE signifies the relative error between the predicted and observed shelf lives. SC and WC indicate the fresh sweet and fresh waxy corn samples, respectively. SO and GL indicate the characteristic sensory qualities of sourness and glossiness, respectively. HB and OR indicates packaging materials of high-barrier nylon/nylon/PP composite film and ordinary nylon/nylon/PP composite film, respectively.

**Table 2:** The validation results of the shelf-life prediction models based on the characteristic indexes of the fresh corn samples.

Sample	Characteristic Index	Storage Method	Temperature (°C)	Predicted Value (Day)	Observed Value (Day)	RE (%)
SC	Color changes	HB	4 °C	123	120	2.5
			25 °C	98	105	6.67
			38 °C	86	90	4.44
		OR	4 °C	113	120	5.83
			25 °C	86	90	4.44
			38 °C	74	75	1.33
	GL	HB	4 °C	76	75	1.33
			25 °C	51	60	15.00
			38 °C	41	60	31.67
		OR	4 °C	54	60	10.00
			25 °C	43	45	4.44
			38 °C	38	45	15.56
	SO	HB	4 °C	228	210	8.57
			25 °C	175	180	2.78
			38 °C	151	150	0.67
		OR	4 °C	212	210	0.95
			25 °C	137	150	8.67
			38 °C	108	105	2.86
WC	Color changes	HB	4 °C	121	120	0.83
			25 °C	106	105	0.95
			38 °C	98	90	8.89
		OR	4 °C	102	105	2.86
			25 °C	77	75	2.67
			38 °C	65	60	8.33
	GL	HB	4 °C	117	105	11.43
			25 °C	74	75	1.33
			38 °C	57	60	5.00
		OR	4 °C	86	90	4.44
			25 °C	75	60	25.00
			38 °C	69	60	15.00
	SO	HB	4 °C	278	210	32.38
			25 °C	194	180	7.78
			38 °C	160	165	3.03
		OR	4 °C	241	210	14.76
			25 °C	147	150	2.00
			38 °C	112	120	6.67

Note: RE denotes the relative error between the predicted and observed shelf life values. SC and WC indicate the fresh sweet and waxy corn samples, respectively. SO and GL indicate the characteristic sensory qualities of sourness and glossiness, respectively. HB and OR indicate the packaging materials of high-barrier nylon/nylon/PP composite film and ordinary nylon/nylon/PP composite film, respectively.

## Data Availability

The data used to support the findings of this study can be made available by the corresponding author upon request.

## Supplementary Materials

The following supporting information can be downloaded at: https://www.mdpi.com/article/10.3390/foods12030478/s1, Figure S1: Corn flavor (A), fullness (B), stickiness (C), and sweetness (SW) of fresh corn packaged with high-barrier and ordinary composite films at three storage temperatures from 0 to 210 d; Table S1: Reaction rate constant and determination coefficient based on the characteristic indexes of fresh corn samples at different temperatures; Table S2: Changes in the content of soluble sugars in samples stored at 4 °C from 0 to 210 d; Table S3: Changes in the content of soluble sugars in samples stored at 25 °C from 0 to 210 d; Table S4: Changes in the content of soluble sugars in samples stored at 38 °C from 0 to 210 d; Table S5: Changes in the content of starch in samples stored at 4 °C from 0 to 210 d; Table S6: Changes in the content of starch in samples stored at 25 °C from 0 to 210 d; Table S7: Changes in the content of starch in samples stored at 38 °C from 0 to 210 d.
