# Supplementary material for: Sensory Evaluation and Model Prediction of Vacuum-Packed Fresh Corn during Long-Term Storage
Source: Foods. 2023 Jan 19;12(3):478. doi: 10.3390/foods12030478 (PMC9914152; doi:10.3390/foods12030478)
Supplement: Supplementary file 1 [file foods-12-00478-s001.zip › foods-2114716-supplementary.pdf]

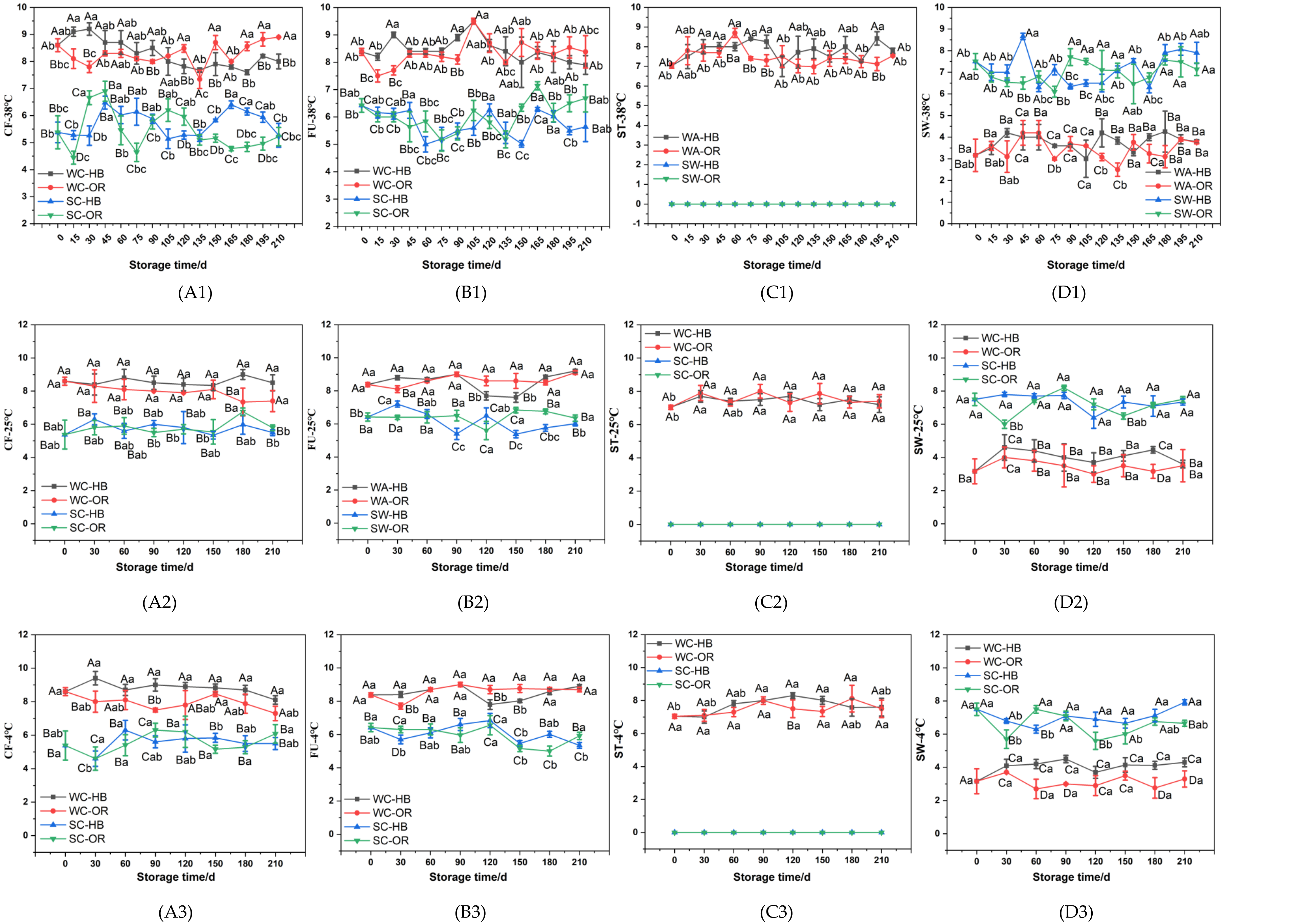

CF-38°C
FU-38°C
ST-38°C
SW-38°C
CF-25°C
FU-25°C
ST-25°C
SW-25°C
CF-4°C
FU-4°C
ST-4°C
SW-4°C
Storage time/d
WC-HB
WC-OR
SC-HB
SC-OR
WA-HB
WA-OR
SW-HB
SW-OR
(A1)
(B1)
(C1)
(D1)
(A2)
(B2)
(C2)
(D2)
(A3)
(B3)
(C3)
(D3)

Figure S1. Corn flavor (A), fullness (B), stickiness (C), and sweetness (SW) of fresh corn packaged with high-barrier and ordinary composite films at three storage temperatures from 0 to 210 days. Different capital letters (A–D) indicate that the significant differences ($p < 0.05$) between different treatments at the same storage time. Different lowercase letters (a–c) indicate that the significant differences ($p < 0.05$) between different storage time for same treatment. Duncan's test was used to analyze differences between groups. WC-HB indicates that the waxy corn packed in high-barrier nylon/nylon/PP composite film. WC-OR indicates that the waxy corn packed in ordinary nylon/nylon/PP composite film. SC-HB indicates that the sweet corn packed in high-barrier nylon/nylon/PP composite film. SC-OR indicates that the sweet corn packed in ordinary nylon/nylo n/PP composite film. CF (A), FU (B), ST(C) and SW(D) indicates that the characteristic sensory quality of corn flavor (CF), fullness (FU), stickiness (ST) and sweetness (SW) respectively.

Table S1. Reaction rate constant and determination coefficient based on the characteristic indexes of fresh corn samples at different temperatures.

Sample	Characteristic index	Storage method	Temperature/℃	Zero-order reaction			First-order reaction		
				equation	k	R^2	equation	k	R^2
SC	color changes	HB	4℃	y = 0.0789x - 1.3346	0.0789	0.983	y = 0.0452e0.0351x	0.0351	0.5915
			25℃	y = 0.0952x + 0.1071	0.0952	0.991	y = 0.0993e0.0326x	0.0326	0.5239
			38℃	y = 0.1132x + 0.3877	0.1132	0.9921	y = 0.4099e0.025x	0.025	0.6229
		OR	4℃	y = 0.0899x - 0.8071	0.0899	0.9952	y = 0.0778e0.0332x	0.0332	0.65
			25℃	y = 0.1026x - 0.1004	0.1026	0.9966	y = 0.0988e0.033x	0.033	0.5627
			38℃	y = 0.1427x + 0.0772	0.1427	0.9984	y = 0.4867e0.0253x	0.0253	0.6938
	GL	HB	4℃	y = -0.0246x + 8.9667	-0.025	0.9785	y = 9.3384e-0.004x	-0.004	0.9755
			25℃	y = -0.031x + 8.7333	-0.031	0.9807	y = 9.4262e-0.006x	-0.006	0.9912
			38℃	y = -0.0472x + 8.3571	-0.047	0.959	y = 14.522e-0.019x	-0.019	0.9184
		OR	4℃	y = -0.0333x + 8.9333	-0.033	0.9374	y = 9.5846e-0.006x	-0.006	0.9606
			25℃	y = -0.0421x + 8.8083	-0.042	0.9873	y = 12.815e-0.014x	-0.014	0.9079
			38℃	y = -0.0476x + 8.2531	-0.048	0.9487	y = 19.686e-0.025x	-0.025	0.8583
	SO	HB	4℃	y = 0.0304x - 0.3292	0.0304	0.989	y = 0.1055e0.0238x	0.0238	0.76
			25℃	y = 0.0346x + 0.1958	0.0346	0.9824	y = 0.1539e0.0232x	0.0232	0.6152
			38℃	y = 0.047x + 0.3504	0.047	0.9724	y = 0.3386e0.0205x	0.0205	0.6243
		OR	4℃	y = 0.0326x + 0.3708	0.0326	0.9877	y = 0.1774e0.0222x	0.0222	0.676
			25℃	y = 0.0442x + 0.4958	0.0442	0.9774	y = 0.2014e0.0234x	0.0234	0.5824
			38℃	y = 0.0662x + 0.6023	0.0662	0.9814	y = 0.3349e0.0291x	0.0291	0.6527
WC	color changes	HB	4℃	y = 0.0599x - 0.6788	0.0599	0.986	y = 0.0534e0.0327x	0.0327	0.5865
			25℃	y = 0.0711x - 0.1254	0.0711	0.9911	y = 0.081e0.0318x	0.0318	0.5599
			38℃	y = 0.0732x + 0.2465	0.0732	0.9866	y = 0.3053e0.024x	0.024	0.6464
		OR	4℃	y = 0.0736x + 0.3154	0.0736	0.9956	y = 0.0978e0.0312x	0.0312	0.5942
			25℃	y = 0.0999x - 0.3346	0.0999	0.9906	y = 0.0877e0.0334x	0.0334	0.5313

		38℃	y = 0.1146x + 5.4905	0.1146	0.8198	y = 0.7329e0.0233x	0.0233	0.3678
GL	HB	4℃	y = -0.0204x + 9.7	-0.02	0.9743	y = 9.9849e-0.003x	-0.003	0.9418
		25℃	y = -0.0281x + 9.6583	-0.028	0.9888	y = 10.277e-0.005x	-0.005	0.9522
		38℃	y = -0.0429x + 9.2275	-0.043	0.9705	y = 11.805e-0.011x	-0.011	0.9353
	OR	4℃	y = 0.0346x + 0.6571	0.0346	0.9889	y = 0.8788e0.0143x	0.0143	0.8648
		25℃	y = 0.0408x + 0.8083	0.0408	0.992	y = 1.1738e0.0114x	0.0114	0.8657
		38℃	y = 0.0431x + 1.8101	0.0431	0.9615	y = 1.8944e0.0097x	0.0097	0.8099
SO	HB	4℃	y = 0.0247x - 0.2913	0.0247	0.9883	y = 0.0338e0.0299x	0.0299	0.6799
		25℃	y = 0.0319x + 0.1754	0.0319	0.9807	y = 0.0566e0.0293x	0.0293	0.5235
		38℃	y = 0.044x + 0.2517	0.044	0.9871	y = 0.1205e0.0271x	0.0271	0.5837
	OR	4℃	y = 0.0281x + 0.1838	0.0281	0.9804	y = 0.0581e0.0284x	0.0284	0.6206
		25℃	y = 0.0441x + 0.4338	0.0441	0.9894	y = 0.0786e0.0295x	0.0295	0.5949
		38℃	y = 0.061x + 0.4718	0.061	0.983	y = 0.09e0.0379x	0.0379	0.5323

Note: K denotes the slope of the fitting curve. R^2 denotes the coefficient of determination of the models, SC and WC indicate the fresh sweet corn samples and the fresh waxy corn samples. SO and GL indicates that the characteristic sensory quality of sourness (SO) and glossiness (GL) respectively. HB and OR indicates that the packaging materials of high-barrier nylon/nylon/PP composite film and ordinary nylon/nylon/PP composite film respectively.

Table S2. Changes in the content of soluble sugars in samples store at 4 °C from 0 to 210 days.

Sample	0d	30d	60d	90d	120d	150d	180d	210d
WC-HB	4.25±0.08^{Ba}	4.61±0.51^{Ba}	3.62±0.06^{Db}	4.40±0.05^{Ba}	3.21±0.04^{Cb}	3.92±0.54^{Ba}	3.21±0.51^{Bb}	4.21±0.41^{Ba}
WC-OR	4.25±0.08^{Ba}	4.55±0.39^{Ca}	4.09±0.09^{Ca}	3.84±0.12^{Cb}	4.01±0.15^{Bab}	4.34±0.17^{Ba}	3.27±0.27^{Bc}	3.91±0.24^{Bab}
SC-HB	6.58±0.12^{Ab}	7.01±0.08^{Aa}	7.02±0.05^{Ba}	6.94±0.14^{Aa}	7.01±0.11^{Aa}	6.91±0.24^{Aab}	6.81±0.34^{Aab}	6.99±0.41^{Aab}
SC-OR	6.58±0.12^{Ab}	7.12±0.10^{Aa}	7.51±0.32^{Aa}	7.12±0.03^{Aa}	6.97±0.15^{Aa}	7.01±0.31^{Aa}	7.13±0.18^{Aa}	7.09±0.95^{Aab}

Note: Different capital letters (A–D) indicate that the significant differences ($p < 0.05$) between different treatments at the same storage time. Different lowercase letters (a–c) indicate that the significant differences ($p < 0.05$) between different storage time for same treatment. Duncan's test was used to analyze differences between groups. WC-HB indicates that the waxy corn packed in high-barrier nylon/nylon/PP composite film. WC-OR indicates that the waxy corn packed in ordinary ny-lon/nylon/PP composite film. SC-HB indicates that the sweet corn packed in high-barrier ny-lon/nylon/PP composite film. SC-OR indicates that the sweet corn packed in ordinary ny-lon/nylon/PP composite film.

Table S3. Changes in the content of soluble sugars in samples store at 25°C from 0 to 210 days.

Sample	0d	30d	60d	90d	120d	150d	180d	210d
WC-HB	4.25±0.08^{Bb}	4.82±0.25^{Ba}	4.09±0.18^{Bb}	4.32±0.35^{Bb}	3.54±0.34^{Cc}	4.19±0.34^{Bbc}	4.01±0.16^{Bb}	4.22±0.05^{Bb}
WC-OR	4.25±0.08^{Ba}	4.29±0.64 ^{Bab}	3.92±0.16^{Bb}	4.02±0.25^{Bab}	3.81±0.29^{Cab}	3.99±0.34^{Bab}	4.02±0.35^{Bab}	4.15±0.21^{Bab}
SC-HB	6.58±0.12^{Ab}	7.19±0.26^{Aab}	7.29±0.10^{Aa}	6.80±0.24^{Ab}	6.72±0.13^{Bb}	6.81±0.42^{Aab}	6.72±0.14^{Ab}	6.88±0.24^{Ab}
SC-OR	6.58±0.12^{Ab}	7.30±0.37^{Aab}	7.48±0.23^{Aa}	7.12±0.23^{Aab}	7.21±0.08^{Aa}	6.75±0.27^{Ab}	6.65±0.23^{Ab}	6.57±0.51^{Ab}

Note: Different capital letters (A–D) indicate that the significant differences ($p < 0.05$) between different treatments at the same storage time. Different lowercase letters (a–c) indicate that the significant differences ($p < 0.05$) between different storage time for same treatment. Duncan's test was used to analyze differences between groups. WC-HB indicates that the waxy corn packed in high-barrier nylon/nylon/PP composite film. WC-OR indicates that the waxy corn packed in ordinary ny-lon/nylon/PP composite film. SC-HB indicates that the sweet corn packed in high-barrier ny-lon/nylon/PP composite film. SC-OR indicates that the sweet corn packed in ordinary ny-lon/nylon/PP composite film.

Table S4. Changes in the content of soluble sugars in samples store at 38°C from 0 to 210 days.

Sample	WC-HB	WC-OR	SC-HB	SC-OR
0d	4.25±0.08^{Bc}	4.25±0.08^{Ba}	6.58±0.12^{Ac}	6.58±0.12^{Ac}
15d	4.55±0.37^{Babc}	4.25±0.42^{Ba}	6.84±0.29^{Aab}	6.91±0.57^{Aabc}
30d	5.02±0.19^{Ba}	4.67±0.09^{Ca}	6.94±0.29^{Aab}	7.06±0.55^{Aabc}
45d	4.49±0.13^{Bbc}	4.51±0.09^{Ba}	7.49±0.35^{Aa}	7.15±0.21^{Aabc}
60d	4.37±0.05^{Cb}	4.68±0.09^{Ba}	7.17±0.31^{Aab}	6.94±0.11^{Ab}
75d	4.12±0.13^{Bcd}	3.93±0.20^{Bbc}	7.15±0.07^{Aa}	7.32±0.08^{Aab}
90d	4.01±0.06^{Cd}	4.21±0.18^{Ca}	7.23±0.14^{Aa}	6.88±0.09^{Bb}
105d	4.05±0.13^{Bcd}	3.55±0.37^{Bc}	7.17±0.50^{Aab}	6.97±0.24^{Ab}
120d	3.21±0.34^{Ce}	4.21±0.15^{Bab}	6.82±0.31^{Aab}	7.13±0.55^{Aabc}
135d	4.47±0.17^{Bb}	4.33±0.17^{Ba}	6.67±0.18^{Abc}	6.58±0.42^{Abc}
150d	4.57±0.24^{Bbc}	4.29±0.21^{Ba}	7.11±0.57^{Aab}	7.01±0.91^{Aabc}
165d	4.14±0.14^{Bcd}	4.33±0.09^{Ba}	6.97±0.15^{Aab}	6.61±0.72^{Aabc}
180d	4.23±0.19^{Bbc}	4.51±0.64^{Ba}	6.79±0.68^{Aabc}	6.83±0.49^{Aabc}
195d	4.17±0.05^{Bcd}	3.90±0.30^{Bab}	7.02±0.51^{Aab}	6.89±0.87^{Aabc}
210d	4.08±0.12^{Bcd}	4.14±0.21^{Bab}	6.99±0.34^{Aab}	6.84±0.62^{Aabc}

Note: Different capital letters (A–C) indicate that the significant differences ($p < 0.05$) between different treatments at the same storage time. Different lowercase letters (a–d) indicate that the significant differences ($p < 0.05$) between different storage time for same treatment. Duncan's test was used to analyze differences between groups. WC-HB indicates that the waxy corn packed in high-barrier nylon/nylon/PP composite film. WC-OR indicates that the waxy corn packed in ordinary ny-lon/nylon/PP composite film. SC-HB indicates that the sweet corn packed in high-barrier ny-lon/nylon/PP composite film. SC-OR indicates that the sweet corn packed in ordinary ny-lon/nylon/PP composite film.

Table S5. Changes in the content of starch in samples store at 4℃ from 0 to 210 days.

Sample	0d	30d	60d	90d	120d	150d	180d	210d
WC-HB	36.74±0.99^{Aa}	32.18±0.64^{Bc}	32.62±0.60^{Bbc}	31.40±0.58^{Ac}	32.67±0.17^{Bbc}	33.27±0.59^{Bb}	36.24±0.92^{Aa}	37.22±0.25^{Aa}
WC-OR	36.74±0.99^{Aab}	35.21±0.47^{Aab}	36.18±0.29^{Aa}	29.84±1.12^{Ad}	34.16±0.45^{Ac}	36.14±0.93^{Aab}	35.27±0.62^{Ab}	35.27±0.47^{Bb}
SC-HB	6.39±0.30^{Ba}	6.49±0.57^{Ca}	6.22±0.54^{Ca}	6.34±0.14^{Ba}	6.33±0.99^{Ca}	6.61±0.31^{Ca}	6.29±0.58^{Ba}	6.43±0.31^{Ca}
SC-OR	6.39±0.30^{Ba}	5.95±0.65^{Ca}	6.12±0.61^{Ca}	6.22±0.08^{Ba}	6.27±0.21^{Ca}	6.24±0.18^{Ca}	6.16±0.81^{Ba}	6.39±0.94^{Ca}

Note: Different capital letters (A–C) indicate that the significant differences ($p < 0.05$) between different treatments at the same storage time. Different lowercase letters (a–d) indicate that the significant differences ($p < 0.05$) between different storage time for same treatment. Duncan's test was used to analyze differences between groups. WC-HB indicates that the waxy corn packed in high-barrier nylon/nylon/PP composite film. WC-OR indicates that the waxy corn packed in ordinary ny-lon/nylon/PP composite film. SC-HB indicates that the sweet corn packed in high-barrier ny-lon/nylon/PP composite film. SC-OR indicates that the sweet corn packed in ordinary ny-lon/nylon/PP composite film.

Table S6. Changes in the content of starch in samples store at 25℃ from 0 to 210 days.

Sample	0d	30d	60d	90d	120d	150d	180d	210d
WC-HB	36.74±0.99^{Aab}	33.21±0.21^{Ac}	30.71±0.82^{Ad}	30.95±0.16^{Ad}	33.59±0.34^{Ac}	33.25±0.27^{Bc}	36.24±0.57^{Ab}	37.22±0.25^{Aa}
WC-OR	36.74±0.99^{Aa}	32.99±1.01^{Ab}	30.23±0.11^{Ac}	29.52±0.71^{Bc}	34.27±1.11^{Ab}	36.91±0.73^{Aa}	34.27±0.99^{Bb}	35.56±1.24^{Bab}
SC-HB	6.39±0.30^{Ba}	6.54±0.98^{Ba}	6.17±0.20^{Ba}	6.23±0.13^{Ca}	6.26±0.08^{Ba}	6.19±0.11^{Ca}	6.08±0.61^{Ca}	6.55±0.32^{Ca}
SC-OR	6.39±0.30^{Ba}	6.57±0.39^{Ba}	6.28±0.32^{Ba}	6.18±0.15^{Ca}	6.40±0.13^{Ba}	6.51±0.71^{Ca}	6.41±0.37^{Ca}	6.47±0.46^{Ca}

Note: Different capital letters (A–C) indicate that the significant differences ($p < 0.05$) between different treatments at the same storage time. Different lowercase letters (a–d) indicate that the significant differences ($p < 0.05$) between different storage time for same treatment. Duncan's test was used to analyze differences between groups. WC-HB indicates that the waxy corn packed in high-barrier nylon/nylon/PP composite film. WC-OR indicates that the waxy corn packed in ordinary ny-lon/nylon/PP composite film. SC-HB indicates that the sweet corn packed in high-barrier ny-lon/nylon/PP composite film. SC-OR indicates that the sweet corn packed in ordinary ny-lon/nylon/PP composite film.

Table S7. Changes in the content of starch in samples store at 38°C from 0 to 210 days.

Sample	WC-HB	WC-OR	SC-HB	SC-OR
0d	36.74±0.99^{Aa}	36.74±0.99^{Aa}	6.39±0.30^{Ba}	6.39±0.30^{Ba}
15d	35.21±0.51^{Aab}	33.78±0.61^{Bbc}	6.56±1.08^{Ca}	5.95±2.15^{Ca}
30d	31.28±0.89^{Ad}	32.12±0.91^{Ac}	6.06±0.84^{Ba}	6.07±0.99^{Ba}
45d	30.94±0.35^{Ad}	31.15±0.11^{Ad}	5.94±0.05^{Ba}	6.15±0.14^{Ca}
60d	30.71±0.25^{Ad}	29.71±0.82^{Ad}	6.17±0.13^{Ba}	6.14±0.11^{Ba}
75d	31.35±0.46^{Ad}	29.93±0.39^{Bd}	6.19±0.07^{Ca}	6.17±0.03^{Ca}
90d	29.15±1.61^{Bd}	30.43±0.17^{Ad}	6.08±0.08^{Ca}	5.98±0.10^{Ca}
105d	36.25±1.01^{Aa}	33.15±0.92^{Bbc}	6.21±0.37^{Ca}	6.37±0.53^{Ca}
120d	33.67±0.45^{Ac}	32.37±0.71^{Bc}	6.32±0.13^{Ca}	6.13±0.27^{Ca}
135d	33.24±0.92^{Ac}	32.07±0.34^{Ac}	6.29±0.42^{Ba}	6.38±0.37^{Ba}
150d	31.27±0.37^{Ad}	31.07±0.16^{Ad}	6.31±0.88^{Ba}	6.27±0.17^{Ba}
165d	29.92±0.61^{Bd}	33.27±0.57^{Abc}	6.30±0.55^{Ca}	6.27±0.27^{Ca}
180d	31.51±0.44^{Ad}	32.54±0.95^{Abc}	6.23±0.31^{Ba}	6.31±0.09^{Ba}
195d	31.27±0.54^{Ad}	30.90±0.42^{Ad}	6.01±0.24^{Ba}	6.18±0.30^{Ba}
210d	34.16±0.84^{Abc}	34.25±0.82^{Ab}	6.33±0.76^{Ba}	6.26±0.21^{Ba}

Note: Different capital letters (A–C) indicate that the significant differences ($p < 0.05$) between different treatments at the same storage time. Different lowercase letters (a–d) indicate that the significant differences ($p < 0.05$) between different storage time for same treatment. Duncan's test was used to analyze differences between groups. WC-HB indicates that the waxy corn packed in high-barrier nylon/nylon/PP composite film. WC-OR indicates that the waxy corn packed in ordinary ny-lon/nylon/PP composite film. SC-HB indicates that the sweet corn packed in high-barrier ny-lon/nylon/PP composite film. SC-OR indicates that the sweet corn packed in ordinary ny-lon/nylon/PP composite film.
